# Silencing of lncRNA XIST inhibits non-small cell lung cancer growth and promotes chemosensitivity to cisplatin

**DOI:** 10.18632/aging.102673

**Published:** 2020-03-25

**Authors:** Xiaohui Xu, Xiaoyun Zhou, Zhenju Chen, Chao Gao, Luo Zhao, Yushang Cui

**Affiliations:** 1 Department of Thoracic Surgery, Peking Union Medical College Hospital, Wangfujing, Dongcheng, Beijing 100730, P.R. China; 2 Beijing 100biotech Co., Ltd., Beijing 100006, China

**Keywords:** lncRNA, XIST, apoptosis, pyroptosis, chemoresistance, NSCLC

## Abstract

Long noncoding RNAs (lncRNAs) play critical roles in tumour progression and metastasis. Emerging evidence indicates that the lncRNA X inactive-specific transcript (XIST) is dysregulated in several tumor types, including non-small cell lung cancer (NSCLC). However, in NSCLC and other cancers the oncogenic mechanism of XIST remains incompletely understood. Here, we confirmed that XIST is upregulated in human NSCLC specimens, and is especially overexpressed in tumors previously treated with cisplatin (cis-diamminedichloroplatinum(II); DDP). In vitro, XIST knockdown inhibited NSCLC cell growth and promoted DDP chemosensitivity by stimulating apoptosis and pyroptosis. Moreover, XIST’s oncogenic effects and ability to promote DDP chemoresistance were largely related to its binding to the TGF-β effector SMAD2, which inhibited its translocation to the nucleus and prevented the transcription of p53 and NLRP3, crucial regulators of apoptosis and pyroptosis, respectively. Using DDP-resistant NSCLC cells, mouse xenograft studies verified the oncogenic function of XIST and its ability to inhibit programmed cell death, thereby mediating DDP chemoresistance. These findings suggest that XIST expression may serve as a novel biomarker to predict DDP treatment efficacy, and may help in the design of new therapies to circumvent DDP chemoresistance in NSCLC and other tumor types.

## INTRODUCTION

Lung cancer is the leading cause of cancer-related deaths in China and around the world. Non-small cell lung cancer (NSCLC) accounts for more than 85% of lung cancer cases and bears the highest mortality rate. Despite substantial therapeutic progress the prognosis of lung cancer remains poor, with a 5-year survival rate lower than 18%. Due to the lack of early diagnostic biomarkers, lung cancer is detected in the majority of the cases in advanced stages: more than 50% of NSCLC patients are diagnosed with locally advanced (stage III) or metastatic (stage IV) disease [1]. Thus, developing novel molecular strategies for early diagnosis is of great importance to improve the overall prognosis of NSCLC patients.

Long noncoding RNAs (lncRNAs) constitute a large class of RNA transcripts greater than 200 nucleotides in length. They are the most abundant type of non-protein-coding RNAs and are involved in numerous physiological and pathological processes, including regulation of gene expression, genomic imprinting, chromatin organization, immune regulation, viral pathogenesis, and oncogenesis [2–7]. A large number of lncRNAs has been identified to date; however, our understanding of lncRNA functions remains vastly incomplete. X inactive-specific transcript (XIST) is a lncRNA encoded by the XIST gene responsible for X-chromosome inactivation in mammals. It was proposed that XIST may exert its biological effects on gene expression by altering the stability of heterochromatin [8–10].

Considerable evidence indicates that XIST plays crucial roles in the differentiation, proliferation, and genome maintenance in human cells. Most studies to date focused on the function of XIST in human cancers, i.e. colorectal cancer [11], uveal NSCLC [12], hepatocellular carcinoma [13, 14], and breast cancer [15, 16]. Knockdown of XIST in NSCLC cells was reported to inhibit TGF-β-induced epithelial-mesenchymal transition and cell migration and invasion through the miR-367/miR-141-ZEB2 axis [17]. Another study revealed that XIST knockdown may suppress NSCLC proliferation and invasion by acting as a miR-186-5p sponge, and indicated the presence of a novel XIST-miR-186-5p regulatory axis in NSCLC [18]. In addition, XIST promoter demethylation was identified as a tissue biomarker for testicular germ cell tumors and spermatogenesis quality [19].

Disruption of the normal apoptotic program has shown to contribute to chemoresistance in many cancer types. In recent years, an alternative form of programmed cell death, i.e. caspase-1 dependent pyroptosis, emerged as a general innate immune effector mechanism in vertebrates [20]. Pyroptosis is a lytic, pro-inflammatory form of cell death featuring cell swelling, pore formation, and “bubbling” and damage of the plasma membrane [21] triggered by numerous pathological conditions, for instance, stroke, myocardial infarction, and cancer [22].

The aim of the present study was to investigate XIST functions, and to specifically assess its role in the development of cisplatin (DDP) resistance, in NSCLC. After verification of XIST overexpression in clinical NSCLC samples, especially DDP-treated specimens, as well as in human NSCLC cell lines, we conducted gain- and loss-of-function studies and co-expression analyses to identify XIST’s binding partners and further elucidate its oncogenic effect. Our data strongly suggest that XIST modulates TGF-β signaling by directly interacting with SMAD2, which impacts on apoptosis, DDP-mediated pyroptosis, and resistance to DDP in NSCLC cells.

## RESULTS

### LncRNA XIST is upregulated in NSCLC

**Figure 1:**
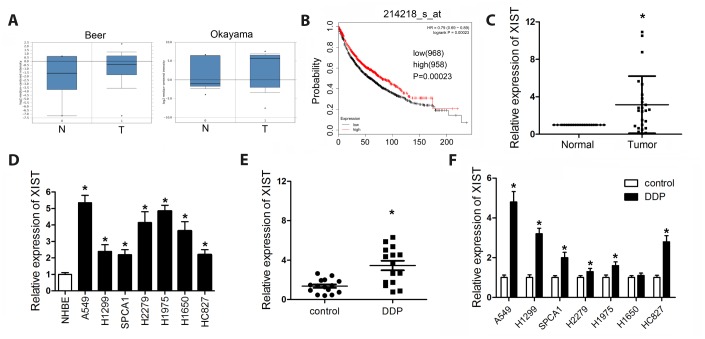
**XIST is downregulated in NSCLC tissues and cell lines.** (**A**) ONCOMINE analysis of XIST expression in clinical tumour samples and adjacent normal tissues. (**B**) KM plotter survival analysis showing the relationship between XIST expression and NSCLC prognosis. XIST expression levels detected by qPCR in (**C**) NSCLC samples and normal tissues, (**D**) NSCLC cells and normal lung epithelial cells, (**E**) NSCLC specimens, and (**F**) untreated or DDP-treated NSCLC cells. * < 0.05 vs control, normal, or NHBE group.

XIST expression levels were investigated in NSCLC microarray datasets retrieved from the ONCOMINE® cancer profiling database (http://www.oncomine.org). The analysis revealed significant upregulation of XIST in NSCLC samples (Figure 1A), and negative correlation between XIST expression and patient survival was detected in the KM plotter database (Figure 1B). Using qPCR, we next assessed XIST expression in human NSCLC and adjacent normal tissue samples, NSCLC cell lines, and normal human lung epithelial cells. Results showed that XIST is significantly upregulated both in clinical NSCLC samples and in NSCLC cell lines (Figure 1C and 1D). Since XIST was reported to be involved in NSCLC chemoresistance to DDP [23], we evaluated the expression of XIST in specimens from patients with or without previous DDP treatment, and in NSCLC cells exposed or not to DDP. We found that XIST levels were more elevated in the tumours of patients who had received DDP treatment, compared to patients not treated with DDP. Consistently, XIST expression was also promoted by DDP treatment in NSCLC cells (Figure 1E and 1F).

### XIST knockdown inhibits proliferation and colony formation and induces apoptosis in NSCLC cell lines

**Figure 2:**
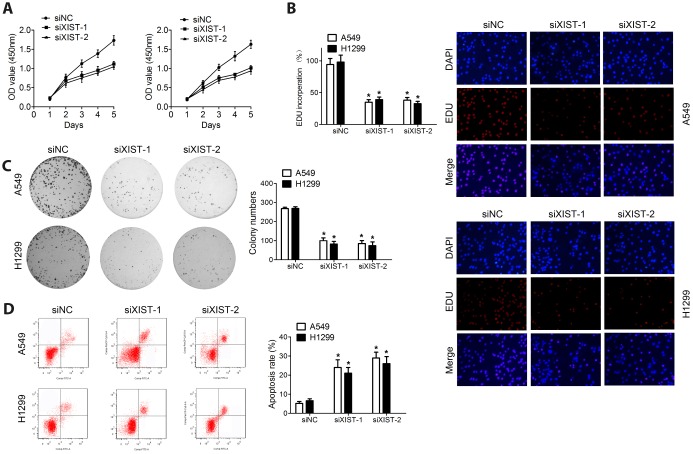
**XIST knockdown inhibits proliferation and colony formation in NSCLC cell lines.** Proliferation of NSCLC cells measured through (**A**) MTT assay and (**B**) EDU staining. (**C**) Colony formation assay results. (**D**) Apoptosis detection by annexin V/PI staining and flow cytometry. * < 0.05 vs si-nc group.

The observed upregulation of XIST in NSCLC samples and cell lines may suggest its involvement in malignant progression. Thus, a siRNA directed against the XIST transcript (si-XIST) was used to knockdown XIST expression, and *in vitro* functional studies, including proliferation, colony formation, and apoptosis analyses, were performed to explore the biological effects of XIST in NSCLC cells. Both MTT assay and EDU staining results revealed that XIST knockdown dramatically suppressed proliferation (Figure 2A and 2B). Accordingly, colony formation ability in cultured NSCLC cells was also inhibited after XIST knockdown (Figure 2C). Interestingly, the growth arrest induced by XIST downregulation was accompanied by induction of apoptosis in both A549 and H1299 cells (Figure 2D).

### XIST knockdown promotes sensitivity to DDP in NSCLC cells

**Figure 3:**
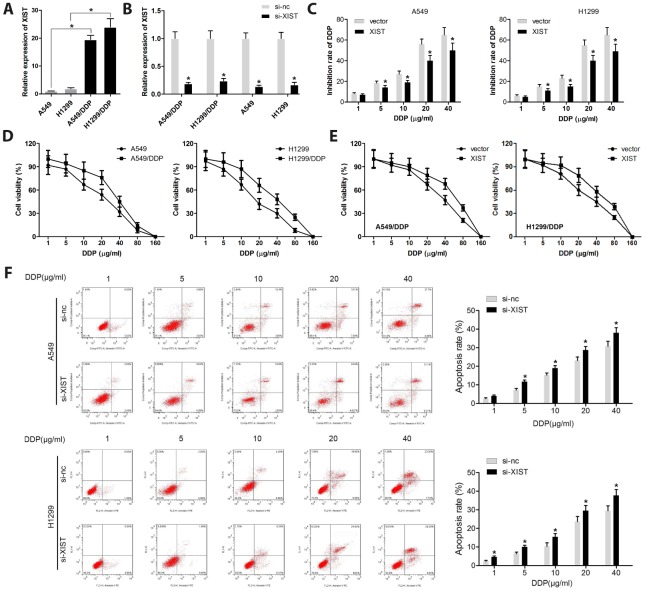
**XIST knockdown restores sensitivity of NSCLC cells to DDP.** (**A**, **B**) XIST expression levels analyzed by qPCR in normal or DDP-resistant NSCLC cells transfected with si-XIST or si-nc (control siRNA). (**C**) Cell proliferation analysis (MTT) results and quantification of DDP inhibition in A549 and H1299 cells. (**D**) Viability assay results for NSCLC cells treated with various concentrations of DDP. (**E**) Viability assay results for XIST-overexpressing A549/DDP and H1299/DDP cells treated with various concentrations of DDP. (**F**) Apoptosis analysis of XIST knockdown effects in NSCLC cells exposed to DDP. * < 0.05 vs si-nc group.

XIST expression has been reported to contribute to the resistance to chemotherapeutic drugs in various types of cancers [24]. Thus, we explored whether XIST is involved in the chemoresistance of NSCLC cells to DDP We found that XIST was overexpressed in DDP-resistant A549 (A549/DDP) and H1299 (H1299/DDP) cells, compared to their DPP-naïve parent cells (Figure 3A). Results of qPCR analyses confirmed that si-XIST transfection markedly inhibited the expression of XIST in A549, H1299, A549/DDP, and H1299/DDP cells (Figure 3B). The MTT assay showed that XIST knockdown significantly inhibited DDP resistance in A549 and H1299 cells (Figure 3C). We verified that under similar DPP concentrations, A549/DDP cells have a higher viability than control A549 cells (Figure 3D), and that XIST overexpression inhibited the chemosensitivity to DPP in A549/DDP and H1299/DDP cells (Figure 3E).

Given that apoptosis escape mechanisms are involved in cancer chemoresistance [25], we evaluated apoptosis in A549 and H1299 cells exposed to various concentrations of DDP. Results revealed that XIST knockdown promoted apoptosis in parent A549 and H1299 cells, and in A549/DDP and H1299/DDP cells treated with DDP. These data indicate that XIST acts as a pro-survival factor in cultured NSCLC cells, and that DDP chemosensitivity can be restored by XIST silencing in our DDP-resistant NSCLC cell lines (Figure 3F).

### XIST interacts with SMAD2 and inhibits its translocation to the cell nucleus

**Figure 4:**
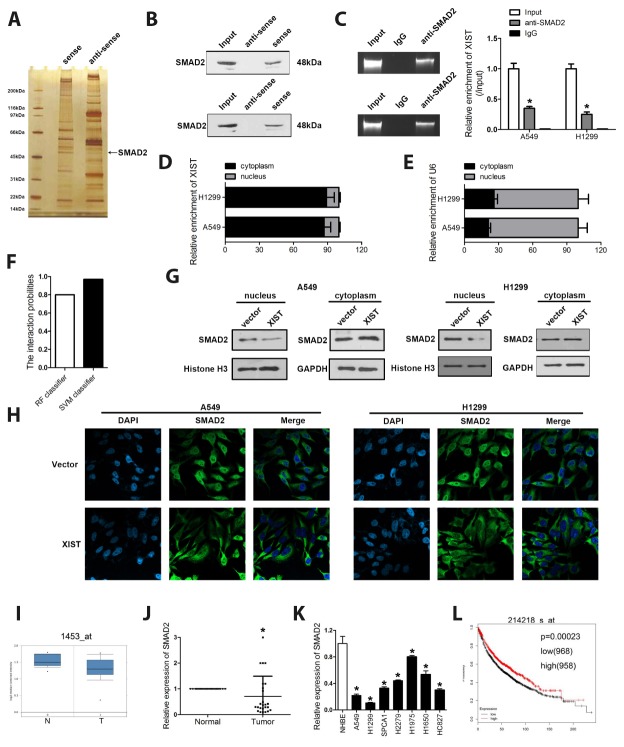
**XIST interacts with SMAD2 and inhibits its translocation to the cell nucleus.** (**A**) RNA pulldown, SDS gel silver staining, and mass spectrometry identified SMAD2 as a binding partner of XIST. (**B**) RNA pulldown and (**C**) RIP assay results demonstrating the XIST/SMAD2 interaction. (**D**, **E**) Cytoplasmic and nuclear expression of XIST detected by qPCR in A549 and H1299 cells. (**G**) Western blot analysis of XIST and SMAD2 expression in cytoplasmic and nuclear cell fractions. (**F**) The score indicating the possibility of the combination between XIST and SMAD2. (**H**) Immunofluorescence assay showing the sub-cellular localization of XIST and SMAD2. (**I**) ONCOMINE analysis of the expression of SMAD2 in clinical NSCLC samples and adjacent normal tissues. XIST expression detected by qPCR in (**J**) NSCLC samples and matched normal tissues and (**K**) NSCLC cells. (**L**) KM plotter survival analysis showing the relationship between SMAD2 expression and NSCLC prognosis. * < 0.05 vs IgG, normal or NHBE group.

The molecular mechanisms underlying the effects of lncRNAs are complex. LncRNAs can sponge miRNAs, directly target mRNAs to alter their translation, or even encode short peptides to perform their functions [26]. We performed RNA pulldown, SDS-PAGE and silver staining, mass spectrometry, and RNA immunoprecipitation (RIP) assays to investigate potential XIST-interacting proteins. These assays indicated that SMAD2 is a potential XIST target (Figure 4A–4C). Since differential localization of lncRNAs may reflect different mechanisms of action (Kopp and Mendell 2018), we assessed XIST’s cellular sub-localization in A549 and H1299 cells using qPCR. Results showed that XIST localizes mainly in the cytoplasm (Figure 4D and 4E). Bioinformatics analysis was performed and indicated a high possibility of the combination between XIST and SMAD2 (Figure 4F). In addition, cytoplasmic and nuclear proteins were separated to detect XIST and SMAD2 levels by western blot. The results revealed that XIST overexpression decreased SMAD2 expression in the nucleus without remarkably changing its cytoplasmic abundance, suggesting decreased nuclear translocation of SMAD2 (Figure 4G). These results were verified by immunofluorescence staining (Figure 4H).

### SMAD2 is downregulated in clinical NSCLC samples and cell lines

To investigate the relevance of the SMAD2/XIST interaction in NSCLC, we first explored the expression of SMAD2 in human NSCLC specimens and in NSCLC cell lines. Our initial ONCOMINE analysis showed that SMAD2 expression (assayed by two different probes) was decreased in NSCLC tissues, compared to matched controls (Figure 4I). Accordingly, a decrease in SMAD2 mRNA levels in NSCLC samples and cells, relative to adjacent normal tissues and lung epithelial cells, respectively, was found by qPCR (Figure 4J and 4K). Suggesting a crucial role for SMAD2 in the progression of NSCLC, survival analysis on the KM plotter database showed a positive correlation between SMAD2 expression and patient survival (Figure 4L).

### SMAD2 silencing rescues the effects of XIST depletion on the proliferation and survival of NSCLC cells

**Figure 5:**
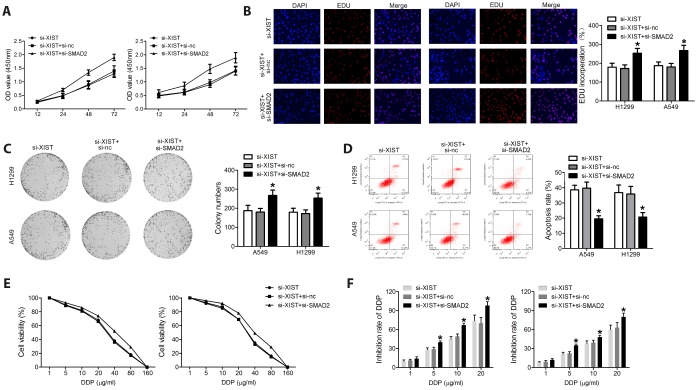
**SMAD2 knockdown rescues the effect of XIST inhibition on the proliferation and survival of NSCLC cells.** Proliferation of NSCLC cells evaluated by (**A**) MTT assay and (**B**) EDU staining. (**C**) Colony formation assay results. (**D**) Apoptosis detection by annexin V/PI staining and flow cytometry. (**E**) Cell viability under different concentrations of DDP was evaluated. (**F**) After transfection, the inhibition rate of DDP on NSCLC cells was assessed. * < 0.05 vs si-XIST group.

To verify whether SMAD2 silencing negates the effects of XIST knockdown on NSCLC cell proliferation and survival, we knocked down both XIST and SMAD2 using specific siRNAs. We found that the effects of XIST knockdown on NSCLC cell proliferation, colony formation and apoptosis were substantially reversed by SMAD2 deletion (Figure 5A–5F).

### DDP induces pyroptosis of NSCLC cells and this effect is potentiated by XIST knockdown

**Figure 6:**
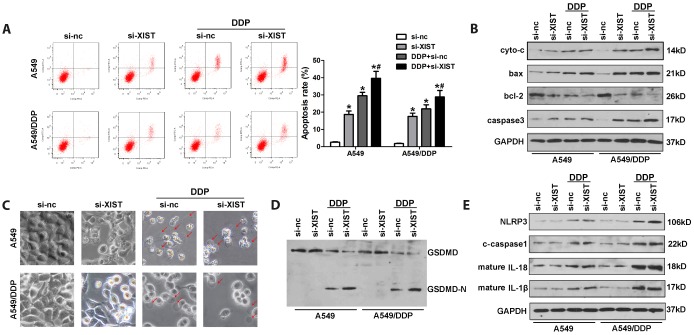
**Induction of pyroptosis by DDP in NSCLC cells and facilitatory role of XIST.** (**A**) Analysis of apoptosis by annexin V/PI staining and flow cytometry in NSCLC cells. (**B**) Western blot detection of apoptosis-related proteins. (**C**) Morphological changes in si-XIST-transfected, DDP-treated NSCLC cells. (**D**, **E**) Western blot analysis of pyroptosis-related proteins. * < 0.05 vs si-nc group, ^#^ < 0.05 vs DDP + si-nc group.

Pyroptosis is an alternative form of programmed cell death that has attracted considerable attention due to its regulatory effect on cancer development and chemoresistance. To evaluate if pyroptosis is involved in NSCLC chemoresistance to DDP, we assessed morphological and molecular markers of apoptosis and pyroptosis in cultured cells. Apoptosis was detected by both annexinV/PI flow cytometry and analysis of apoptosis-related proteins, including cytochrome c (cyto-c), bax, bcl-2, and caspase-3, by western blot. Pyroptosis was investigated by morphological observation and evaluation of pyroptosis-related proteins such as GSDME, GADMD, NLRP3, caspase-1, IL-18, and IL-1β using western blot. The results confirmed that XIST knockdown promoted apoptosis in NSCLC cells (Figure 6A). Accordingly, western blot analysis indicated that XIST knockdown promoted bax, cyto-c, and caspase-3 expression, and inhibited the expression of bcl-2 (Figure 6B). As shown in Figure 6C, these changes were concomitant with decreased proliferation of NSCLC cells. Interestingly, we found morphological evidence that DDP treatment also induced pyroptosis in both A549 and A549/DDP cells (Figure 6C). Accordingly, elevated levels of NLRP3, caspase1, IL-1β, and IL-18, as well as cleaved GSDMD were detected by western blot in DDP-treated cells (Figure 6D, 6E).

### XIST knockdown inhibits NSCLC xenograft growth

**Figure 7:**
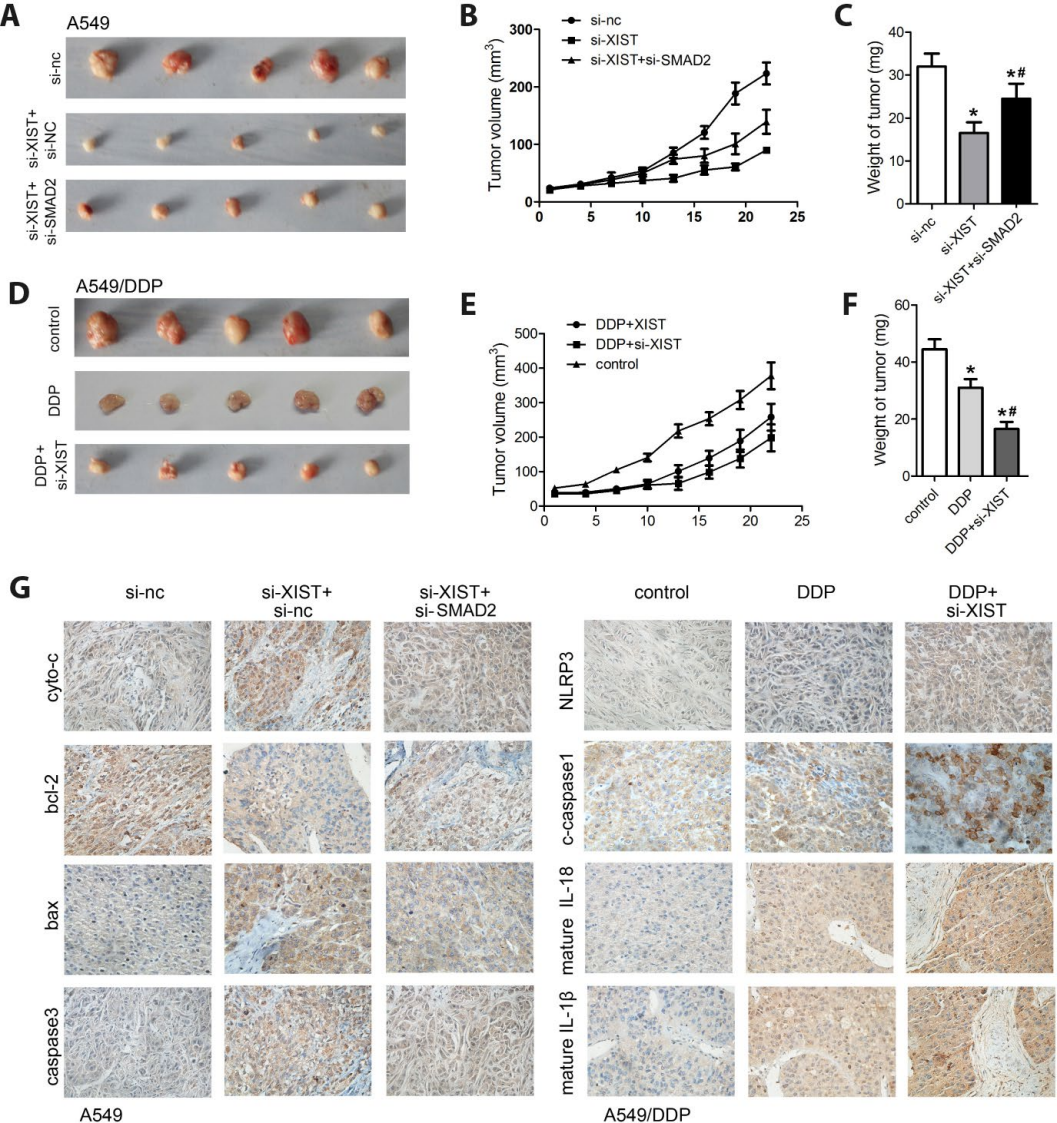
**XIST silencing inhibits NSCLC growth *in vivo*.** (**A**) A xenograft nude mouse model was established using A549 cells transfected with si-XIST, si-SMAD2, or their combination. (**B**) Tumor volume measurements. (**C**) Tumor weight measurements. (**D**) A xenograft nude mouse model was established using A549/DDP cells transfected with si-XIST and subjected to DDP treatment. (**E**) Tumor volume measurements. (**F**) Tumor weight measurements. (**G**) IHC analysis of the expression of apoptosis (cyto-c, bax, bcl-2, and caspase-3) and pyroptosis (NLRP3, caspase-1, IL-1b, and IL-18) markers in excised tumor samples. * < 0.05 vs control or si-nc group, ^#^ < 0.05 vs DDP or si-XIST group.

To evaluate the tumorigenic potential of XIST *in vivo*, a xenograft nude mouse model was established by subcutaneous injection of A549 cells (Figure 7A). Compared with the control group, tumors formed by A549 cells with stable expression of a XIST shRNA vector demonstrated decreased growth kinetics (Figure 7B and 7C). Consistent with the above *in vitro* studies, concomitant deletion of SMAD2 reversed this effect. Then, we investigated the effect of XIST silencing on the growth of A549/DDP cells in DDP-treated mice (Figure 7A–7C). Compared to control, DDP notably inhibited the growth of A549/DDP cells, XIST knock down promoted the chemosensitivity of A549/DDP cells (Figure 7D and 7F). Following tumor excision, IHC analysis was used to determine tumoral expression of apoptosis- and pyroptosis-related biomarkers, including NLRP3, IL-18, IL-1β, and caspase-1. The results confirmed that XIST knockdown promoted apoptosis, which could be reversed by SMAD2 depletion in A549 cells, and potentiated DDP-induced pyroptosis in A549/DDP cells (Figure 7G).

### XIST expression regulates SMAD2-dependent transcription of p53 and NLRP3

**Figure 8:**
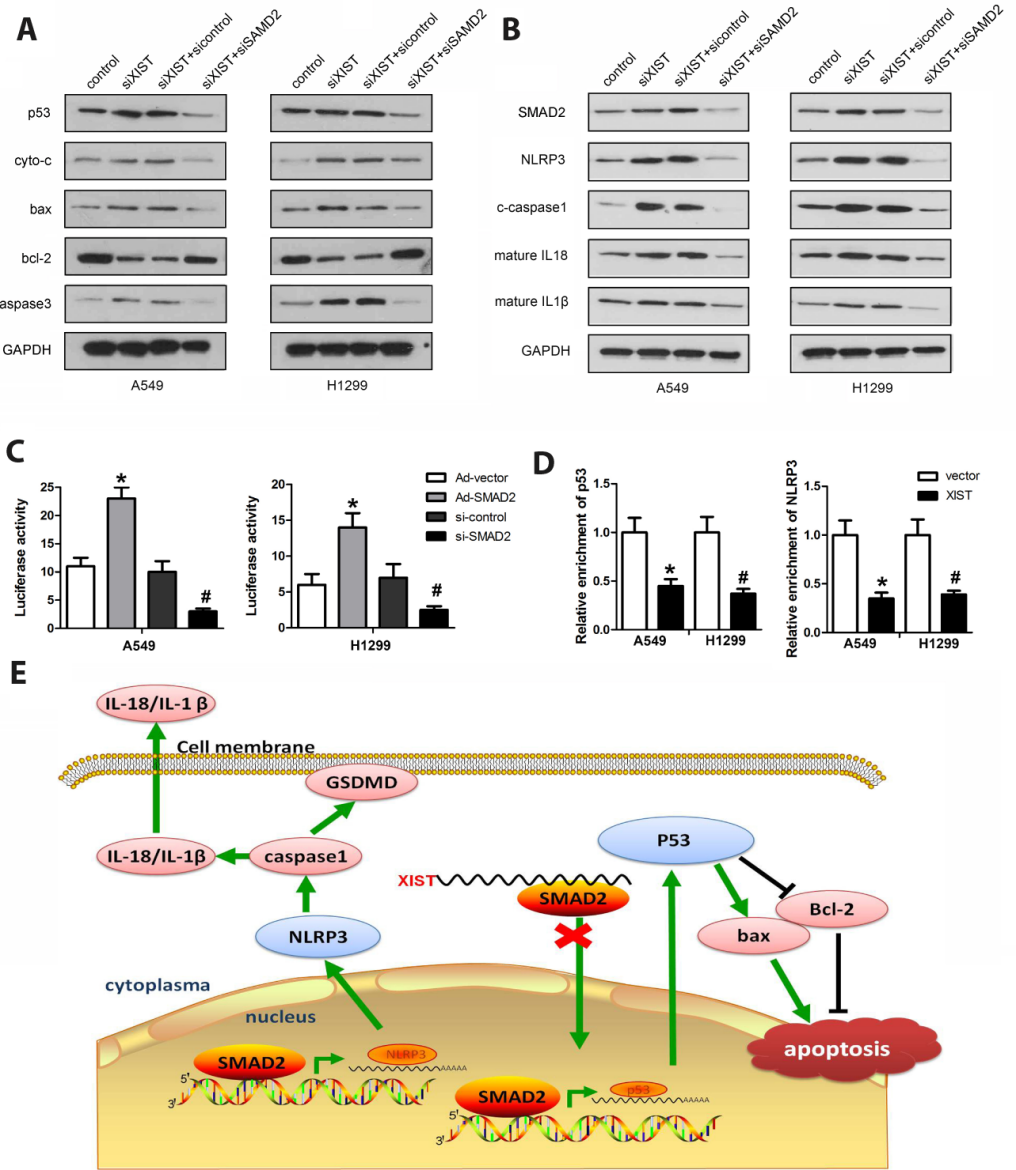
**Co-regulation of apoptosis and pyroptosis pathways by XIST and SMAD2.** (**A**, **B**) Western blot analysis of SMAD2, p53, NLRP3, and apoptosis- and pyroptosis-related proteins in NSCLC cells. (**C**) ChIP assay results indicating reduced interaction of SMAD2 with p53 and NLRP3 following XIST overexpression. (**D**) Luciferase activity assay showing that SMAD2 knockdown reduces the transcription of p53 and NLRP3. (**E**) Schematic diagram depicting the potential mechanism mediating XIST-dependent DDP chemoresistance in NSCLC cells.

To further investigate the effects of XIST and SMAD2 on cell death pathways in NSCLC cells, western blot analysis was conducted to assess the expression of apoptosis- and pyroptosis-related proteins in A549 and H1299 cells transfected with XIST or SMAD2 siRNAs. As shown in Figure 8A and 8B, XIST knockdown led to a significant increase in pro-apoptotic (p53, cyto-3, bax, and caspase-3) and pro-pyroptotic (NLRP3, caspase-1, IL-18, and IL-1β) protein levels, while expression of anti-apoptotic bcl-2 was reduced. In turn, SMAD2 deletion significantly reversed XIST knockdown’s effects. Next, ChIP and luciferase activity assays were performed to evaluate whether SMAD2 can activate the transcription of p53 and NLRP3. We found that SMAD2 effectively interacted with the promoter sequences of p53 and NLRP3, and these interactions were attenuated by XIST (Figure 8C). In turn, luciferase assays showed that SMAD2 decreased the transcription of both p53 and NLRP3 (Figure 8D). These findings revealed that XIST interacts with SMAD2, preventing its nuclear translocation and the transcription of the p53 and NLRP3 genes. The proposed mechanism underlying XIST effects on NSCLC cells is depicted in Figure 8E.

## DISCUSSION

Mounting evidence supports the regulatory role of lncRNAs in cancer progression. The present study confirmed that the lncRNA XIST is upregulated in clinical NSCLC samples, and its expression is further enhanced in cisplatin-resistant tumors and in human NSCLC cells treated with cisplatin (DDP). Importantly, we unmasked a novel interaction with SMAD2 that might critically mediate XIST-mediated resistance to DDP in NSCLC. Hints into the biological effects of XIST on NSCLC had been provided in previous studies. XIST was shown to sponge several miRNAs, such as miR144-3p (Tian et al., 2019), miR-137 [27, 28], miR-367 [29], let-7 [30], and miR-92b [31] and thus regulate the expression of various genes, including Notch-1, PXN, ZEB2, BAG-1, and Smad7. Meanwhile, Fang et al. showed that XIST interacts with EZH2 to suppress transcription of potential targets such as KLF2, and this event is partly responsible for the oncogenic functions of XIST in NSCLC [32].

To extend our understanding of the mechanisms that mediate XIST functions in NSCLC, we performed RNA pulldown to detect possible molecular partners. Mass spectroscopy and RIP assay confirmed that the SMAD2 protein was targeted by XIST. SMAD2 is a prominent effector of TGF-β signaling [33, 34] which plays a central role in the regulation of cell proliferation, differentiation, apoptosis, and development in numerous biological systems [35, 36]. Canonical TGF-β signaling starts with ligand-induced oligomerization of serine/threonine receptor kinases and phosphorylation of the cytoplasmic signalling molecules SMAD2 and SMAD3. These two proteins then form a complex with SMAD4, which translocates into the nucleus and binds SMAD-binding elements (SBE) to activate gene transcription [37, 38]. We found that XIST binds to SMAD2 in the cytosol and inhibits its translocation into the nucleus, which should lead to a reduction in SMAD2-dependent gene transcription. Since XIST depletion was associated with decreased proliferation and survival of NSCLC cells, we focused on potential interactions between SMAD2 and master regulators of programmed cell death pathways. Using CHIP and luciferase reporter activity assays, we revealed that SMAD2 targets the promoters of NLRP3 and p53 and activates the transcription of both genes.

The tumor suppressor p53 is dysregulated in many cancers and its role in tumor progression has been extensively studied. As a key transcription factor, p53 targets multiple genes involved in cell cycle arrest, apoptosis, DNA repair, and angiogenesis [39, 40]. While substantial evidence exists for SMAD2 and p53 crosstalk in gene expression regulation [41], to the best of our knowledge we are the first to demonstrate that SMAD2 can promote the transcription of p53. Our finding that XIST knockdown promoted apoptosis of NSCLC cells, in parallel with increased expression of pro-apoptotic proteins such as cyto-c, bax, and caspase-3, is consistent with XIST-mediated repression of SMAD2 nuclear translocation and inhibition of p53 transcription. However, it remains unclear whether p53 regulates the expression of apoptotic proteins directly or indirectly.

Moreover, we confirmed a similar association between SMAD2 and the promoter of the NLRP3 gene, a key component of the inflammasome complex. This discovery prompted us to focus on pyroptosis, which is morphologically and mechanistically distinct from other forms of cell death [42, 43]. During pyroptosis pores are formed in the cell membrane, which cause water influx and cell swelling resulting in the rupture of the cell membrane. Caspase-1 is a key inductor of pyroptosis, while caspase-3 activation is mainly associated with apoptotic cell death [44]. Research has shown that caspase-1 is not involved in apoptosis, as caspase-1-deficient mice have no defects in the apoptotic program and develop normally [45]. Conversely, apoptotic caspases, including caspase-3, caspase-6, and caspase-8, are not involved in pyroptosis. Substrates of apoptotic caspases, including poly (ADP-ribose) polymerase and inhibitor of caspase-activated DNase (ICAD), do not undergo proteolysis during pyroptosis [45]. Furthermore, loss of mitochondrial integrity and release of cytochrome-c, which can activate apoptotic caspases, do not occur during pyroptosis. However, recent studies have defined a link between pyroptosis and apoptosis which changed our understanding of the relation between them. For instance, caspase-1 was reported to initiate apoptosis in the absence of gasdermin D (GSDMD), which is involved in the bid-caspase-9-caspase-3 axis [46]. Members of the gasdermin family of proteins, which includes GSDMA, GSDMB, GSDMC, GSDMD, and GSDME are critical mediators of pyroptosis [47]. Unlike GSDMD, which is cleaved by inflammatory caspases to induce pyroptosis downstream of inflammasome activation, GSDME is cleaved by caspase-3 and can also induce pyroptosis downstream of apoptosis, thus linking both processes. Moreover, GSDME can potentiate caspase-3/7 activation by targeting the mitochondria to release cytochrome-c, leading to apoptosis. Numerous studies revealed that defective apoptosis is involved in cisplatin chemoresistance in various cancers, including NSCLC [48, 49]. Likewise, pyroptosis failure has also been proved to participate in the progression of chemoresistance in cancers. For instance, the NLRP3 inflammasome is activated by 5-fluorouracil (5-FU) in oral squamous cell carcinoma (OSCC) cells, and mediates chemoresistance by inhibiting apoptosis via ROS/IL-1β upregulation [50, 51]. Combined with the evidence above, our findings suggest that both apoptosis and pyroptosis are involved in the progression of DDP resistance in NSCLC. However, further studies are needed to clarify the specific mechanisms and environmental clues involved in the onset of chemoresistance mediated by the disruption of these forms of programmed cell death.

Although the present results expand our understanding of the mechanisms that mediate DDP resistance in NSCLC cells, several knowledge gaps remain. We showed that XIST knockdown promotes apoptosis of NSCLC cells; however, pyroptosis occurred only after DDP treatment, regardless of whether XIST was silenced or not. These findings would suggest that DDP therapy activates pyroptosis in NSCLC cells, and XIST overexpression prevents this effect. DDP was reported to increase the chemosensitivity of esophageal squamous cell carcinoma both *in vivo* and *in vitro* by inducing bax/caspase-3/GSDME-dependent pyroptosis [52]. To elucidate whether pyroptosis is an independent inducer of apoptosis, we evaluated GSDMD and GSDME expression and found that GSDMD, but not GSDME, was cleaved by DDP treatment. Although the reason for this effect remains to be investigated, this finding would suggest that DDP-induced pyroptosis proceeds independently of apoptosis in NSCLC cells, and suggests a novel mechanism contributing to chemoresistance to DDP.

In summary, the present study revealed new aspects of the oncogenic role of the lncRNA XIST in NSCLC. Our data indicates that XIST can promote proliferation and mediate chemoresistance to DDP in NSCLC by reducing the nuclear transfer of SMAD2, thereby inhibiting apoptosis and pyroptosis by blocking the transcription of p53 and NLRP3 respectively. Our findings provide potential novel biomarkers to assess DDP treatment efficacy and may guide new efforts to prevent or counteract DDP chemoresistance in NSCLC and perhaps other tumor types.

## MATERIALS AND METHODS

### Clinical specimens

A total of 30 NSCLC specimens along with matched, adjacent normal tissue samples were obtained from patients who underwent surgery at Peking Union Medical College Hospital. Sixteen patients had received cisplatin therapy; the remaining 14 patients were not treated with cisplatin. Samples were frozen in liquid nitrogen immediately after resection. The present study was approved by the Ethics Committee of Peking Union Medical College Hospital. Written informed consent was obtained from each patient.

### Cell culture

Non-small cell lung cancer cell lines A549 and H1299 were obtained from the Chinese Academy of Science. A549/DDP (cisplatin-resistant A549 cells) and H1299/DDP (cisplatin-resistant H1299 cells) were purchased from Shanghai Meixuan Biological Science and Technology Co, Ltd. The viability of A549/DDP cells under DDP treatment is significantly higher than that of normal A549 cells subjected to the same DDP treatment. All cells were maintained in RPMI 1640 medium (Gibco, Grand Island, NY, USA) containing 10% FBS with 100U/mL penicillin-streptomycin (Invitrogen, Carlsbad, CA, USA). Cells were cultured at 37°C in 5% CO_2_.

### Transfection and stable cell line generation

SiRNAs targeting XIST and SMAD2 (si-XIST and si-SMAD2) and their negative controls were synthesized by GenePharma (Shanghai, China). For XIST overexpression the XIST sequence was amplified and inserted into a pcDNA3.1 vector; a blank vector was used as control. The pLKO.1 vectors containing shRNA sequences for XIST and SMAD2 were designed and synthesized by Genewiz (Beijing, China). A549 and H1299 cells (5 × 10^5^) were seeded into 6 well plates. After 24 h, the cells were transfected these oligonucleotides (200 nmol/l) or co-transfected with si-XIST and si-SMAD2 using Lipofectamine 2000 (Invitrogen, Carlsbad, CA, USA) according to the manufacturer instructions. Stable cells expressing high levels of sh-XIST and sh-SMAD2 and their negative controls were selected in the presence of 500 μg/ml puromycin.

### Cell viability assay

The 3-(4,5-dimethylthiazol-2-yl)-2,5-diphenyltetrazolium bromide (MTT) assay was used to assess cell viability in A549 and H1299 cells plated in 96-well plates (6 × 10^3^ cells/well). After incubation for 24 h, 0.5% MTT (Sigma, USA) was added to the culture medium at 37°C for 4 h. The supernatant was then removed and DMSO was added into each well. Thereafter, absorption was evaluated at 490 nm using a microplate reader (Bio-Rad, USA).

### Cell proliferation assay

A549 and H1299 cells were supplemented with 45 μM 5-ethynyl-2’-deoxyuridine (EdU, Beyotime, Shanghai, China) for 2 h at 37°C and then fixed in 4% paraformaldehyde for 30 min at room temperature. Then, 1% TritonX-100 was added for permeabilization and the cells were exposed to EdU reaction cocktail (Click-iT EdU microplate assay kit) for 30 min according to the kit instructions. Finally, cell nuclei were stained with 1 mg/ml DAPI (Beyotime, Shanghai, China) for 15 min and the samples observed under a fluorescence microscope (Leica, Wetzlar, Germany).

### Colony formation assay

Cells were treated under the indicated conditions and seeded in 12-well plates (100 cells/well). After incubation for 2 weeks, cell colonies were stained with 0.05% crystal violet (Beyotime, Shanghai, China) and observed under a fluorescence microscope. Colonies containing more than 50 cells were counted.

### Quantitative real-time PCR

Total RNA was extracted using TRIzol reagent (Invitrogen, Carlsbad, CA, USA) according to the manufacturer’s instructions. Total RNA (1 μg) was used as a PCR template and was reverse-transcribed into cDNA using an RNA PCR Kit (Takara, Dalian, China). Quantitative real-time PCR (qPCR) was performed using an iCycler iQ system with iQ SYBR Green Super Mix (Bio-Rad, USA) according to the manufacturer’s instructions. Endogenous U6 small nuclear RNA was used as internal control for miRNA normalization, and GAPDH was used to normalize expression data for mRNAs. Relative gene expression levels were calculated using the (2^-ΔΔCt^) method.

### Western blotting

Protein samples were extracted from A549 and H1299 cells using lysis buffer. Aliquots of 40 μg protein were separated by 10% sodium dodecyl sulfate-polyacrylamide gel electrophoresis (SDS-PAGE) and transferred to polyvinylidene difluoride (PVDF) membranes. The membranes were incubated with 5% skimmed milk in PBS for 2 h to block nonspecific binding, and subsequently probed with primary antibodies overnight at 4°C. After washes with TBST 3 times, the blots were incubated at room temperature for 90 min with horseradish peroxidase-conjugated goat anti-rabbit antibodies. GAPDH was used as internal control. Finally, the membranes were treated with ECL plus reagent (Pierce, Rockford, IL, USA) and an ImageQuant LAS 4000 CCD imager (Fujifilm, Valhalla, NY, USA) was used to visualize protein bands.

### Apoptosis assay

Cultured A549 and H1299 cells were digested with trypsin, washed with cold PBS, and dual-stained using an annexin V FITC/propidium iodide kit according to the manufacturer’s instructions (Beyotime, Shang, China). Apoptotic cells were detected by flow cytometry on a BD FACSCalibur cytometer (Becton Dickinson, NJ, USA).

### RNA pulldown assay

Biotin-labelled XIST probes (sense and antisense) were synthesized by Sangon Biotech (Shanghai, China). Probe-coated beads were generated by incubating the probe with streptavidin-coated beads (Invitrogen) at 25°C for 2 h. A549 and H1299 cells were collected, lysed, and incubated with biotin-labelled probes overnight at 4°C. Then the beads were eluted and RNA-protein complexes purified with TRIzol (Takara, Dalian, China). SMAD2 expression was analyzed by western blot.

### Silver staining

The proteins present in the RNA pulldown material were separated via SDS-PAGE. Gel staining was carried out with a SilverQuest™ silver staining kit (Invitrogen, CA, USA) according to the manufacturer's instructions. Finally, mass spectrometry analysis was performed to evaluate XIST-bound proteins.

### RNA immunoprecipitation (RIP) assay

RIP assay was carried out using an RNA-binding protein immunoprecipitation kit (Millipore, Billerica, MA, USA) according the manufacturer’s instructions. Mouse IgG (Millipore, Billerica, MA, USA) was used as control. Identical amounts of control IgG and SMAD2 antibodies were used (40 μg). The RNAs immunoprecipitated by the SMAD2 antibody were evaluated by qPCR.

### Luciferase activity assay

The 1500 bp promoter regions of p53 and NLRP3 were cloned and inserted into pGL3 vectors (Promega, Madison, WI, USA). Cells were co-transfected with the pRL-CMV plasmid and pGL3-p53 or pGL3-NLRP3 vectors along with siRNAs (si-control or si-SMAD2) using Lipofectamine 2000 (Invitrogen, CA, USA). The luciferase activity assay was performed using a dual-luciferase reporter kit (Dual-Luciferase® Reporter Assay System, Promega, Madison, WI, USA). Luciferase activity was normalized to the activity of Renilla luciferase.

### Chromatin immunoprecipitation (ChIP) assay

ChIP was carried out using an EZ ChIP kit (Millipore, CA, USA). After transfection, A549 and H1299 cells were collected and crosslinked with 1% formaldehyde for 10 min at 37°C. Chromatin was sonicated, incubated and precipitated with anti-SMAD2. The immunoprecipitated DNA fragments were detected by qPCR analysis.

### Immunostaining

Cells were fixed with 4% paraformaldehyde for 20 min, permeabilized with 0.5% Triton X-100 for 15 min, and blocked with 3% BSA for 30 min. Then, the cells were incubated with a primary antibody against SMAD2 (Abcam, Cambridge, England) at 4°C overnight. After washing 3 times, the cells were incubated with an Alexa Fluor-labelled secondary antibody (Invitrogen), stained with DAPI, and observed under a fluorescence microscope.

### In vivo study

Fifteen BALB/c nude male mice (Vital River Laboratory Animal Technology Co., Beijing, China) were randomly divided into six groups. A549 cells with stable expression of sh-XIST, sh-SMAD2, or their combination (Groups 1-3), and A549/DDP cells with stable expression of sh-XIST, sh-RNA control, or their combination (Groups 4-6), were subcutaneously injected (1×10^6^ cells) into the flanks of mice. DDP (50 mg/kg) was intraperitoneally administered to the 3 groups of mice inoculated with A549/DDP cells. Tumour size was measured every two days using a caliper. The mice were sacrificed on day 30 and the tumours were excised. Tumor fractions were alternatively placed in 10% formalin for histological and frozen at -80°C for molecular analyses. Animal experiments were approved by the Ethics Committee of Peking Union Medical College Hospital.

### Immunohistochemistry (IHC)

Sections (5 μm in thickness) were deparaffinized in xylene and rehydrated in graded alcohol series. Hydrogen peroxide (3% in methanol) was used to block endogenous peroxidase activity. After antigen retrieval (heated 10 mM citrate buffer), the slides were incubated with primary antibodies (cytochrome-c, bax, bcl-2, caspase3, NLRP3, caspase1, IL-1β, and IL-18) overnight at 4°C followed by incubation with an HRP-labelled secondary antibody for 2 h at room temperature. A DAB detection system (Dako, Glostrup, Denmark) was used for signal detection according to protocol.

### Statistical analysis

SPSS 20.0 (SPSS Inc., Chicago, IL, USA) and Graph Pad Prism 7.0 (Graph Pad Software, Inc., La Jolla, CA, USA) were used to analyze all data for statistical significance. All data are presented as the mean ± SD. Differences between two means were analyzed by Student’s t-test, and one-way ANOVA was used for multiple group comparisons. *P* < 0.05 was considered significant.
